# Effect of Selenium Nanoparticle Size on IL-6 Detection Sensitivity in a Lateral Flow Device

**DOI:** 10.1021/acsomega.2c07297

**Published:** 2023-02-23

**Authors:** Zoe Bradley, Patrick A. Coleman, Melissa A. Courtney, Sam Fishlock, Joseph McGrath, Therese Uniacke-Lowe, Nikhil Bhalla, James A. McLaughlin, John Hogan, John P. Hanrahan, Ke-Ting Yan, Philip McKee

**Affiliations:** † Environmental Research Institute, Glantreo Ltd., Cork T23 XE10, Ireland; ‡ Department of Chemistry, College of SEFS, University College Cork, Kane Building, Cork T12 YN60, Ireland; § Biopanda Reagents Ltd., Unit 14, Carrowreagh Business Park, Carrowreagh Road, Belfast BT16 1QQ, United Kingdom; ∥ Nanotechnology and Integrated Bioengineering Centre, School of Engineering, University of Ulster, Belfast BT15 1ED, United Kingdom; ⊥ Healthcare Technology Hub, School of Engineering, University of Ulster, Belfast BT15 1ED, United Kingdom; # Department of Chemistry, School of Food and Nutritional Sciences, University College Cork, Level 2 Food Science Building, Cork T12 TP07, Ireland

## Abstract

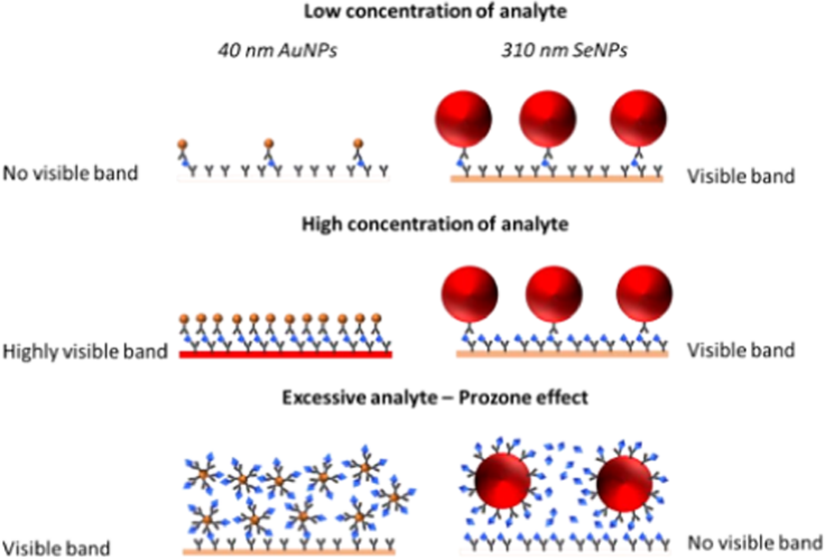

Sepsis is the body’s response to an infection. Existing diagnostic testing equipment is not available in primary care settings and requires long waiting times. Lateral flow devices (LFDs) could be employed in point-of-care (POC) settings for sepsis detection; however, they currently lack the required sensitivity. Herein, LFDs are constructed using 150–310 nm sized selenium nanoparticles (SeNPs) and are compared to commercial 40 nm gold nanoparticles (AuNPs) for the detection of the sepsis biomarker interleukin-6 (IL-6). Both 310 and 150 nm SeNPs reported a lower limit of detection (LOD) than 40 nm AuNPs (0.1 ng/mL compared to 1 ng/mL), although at the cost of test line visual intensity. This is to our knowledge the first use of larger SeNPs (>100 nm) in LFDs and the first comparison of the effect of the size of SeNPs on assay sensitivity in this context. The results herein demonstrate that large SeNPs are viable alternatives to existing commercial labels, with the potential for higher sensitivity than standard 40 nm AuNPs.

## 1 Introduction

Sepsis is the body’s extreme response to an infection. It is a leading cause of mortality globally^1^ due to difficulties in detecting early symptoms and instigating appropriate treatment.^2^ Initial diagnosis tests that take place in primary care settings are limited. Many cases are missed, leading to later-stage detection in hospitals and associated poor outcomes.^3^ Blood cultures are the gold standard for sepsis identification and guiding antibiotic treatment, but long incubation times of up to 72 h^4^ risk the patient developing septic shock and organ failure.^5^

Lateral flow devices (LFDs) have been proposed as an alternative method for sepsis detection at the point-of-care (POC).^6^ LFDs are simple, cost-effective, and portable devices which rapidly detect analytes. They are already used to detect food contaminants,^7^ infectious diseases, and environmental pollutants.^8,9^ The importance of onsite fast testing was highlighted during the SARS-CoV-2 pandemic, where LFDs were used to monitor and prevent viral spread.^10,11^

Interleukin-6 (IL-6) increases within a few minutes of stimulus and has a half-life of 30 min, which is ideal for neonatal sepsis diagnosis.^6^ This work investigates and compares the performance of large selenium nanoparticles (SeNPs) to that of commercial standard 40 nm gold nanoparticles (AuNPs) in the detection of IL-6 in a LFD.

Traditionally, latex nanoparticles (LXNPs) and AuNPs have been the preferred labels for LFDs.^12^ However, both still struggle to match the sensitivity levels of well-established analytical techniques, such as RT-PCR,^13^ ELISA,^14^ and microtube dilution gradient assays,^15^ limiting the applicability of LFDs at POC.^6^ Dyeing of LXNPs,^12,16^ as well as the silver and enzyme surface functionalization of AuNPs,^17,18^ has been used to combat low sensitivity. However, these extra steps increase preparation times and add additional costs.^18−20^ Commercial interest has shifted to develop a new generation of LFD labels which include SeNPs.^21^

Selenium is a metalloid with unique properties, which can be synthesized and functionalized on the nanoscale.^22,23^ Chemical reduction is the preferred synthesis method for SeNPs consistent in size, morphology, and monodispersity.^24−26^ Additionally, SeNPs are stable, easy to functionalize, and cost-effective with a size-dependent optical profile from yellow to red.^22,24^ SeNPs have been utilized in assays for *Escherichia coli*,^27^ pregnancy,^28,29^ and are used commercially for HIV.^30,31^ Among these tests, some report equal or higher sensitivity compared to AuNPs,^32−35^ whereas others report lower sensitivity.^36,37^ Many factors affect label sensitivity including shape, surface chemistry, and size.^38,39^ Using larger NPs can improve sensitivity with lower limits of detection (LODs) at the cost of steric hindrance effects reducing test line visibility at higher concentrations of an analyte.^39,40^ SeNPs commonly employed in LFDs are below 100 nm size.^32,34,36^ While sub-100 nm SeNPs perform well on the small scale, the high centrifugation speeds used for cleaning can cause the formation of Se nanorods, reducing overall monodispersity and preventing commercialization as a product.^41^ Therefore, there is a need to explore the physio-optical properties of large SeNPs, which are a more commercially viable product.

In this investigation, large SeNPs were for the first time synthesized by chemical reduction with size between 150 and 310 nm and successfully used in a LFD. The main objective of this study is to determine the sensitivity of IL-6 using a LFD to compare the effect of SeNPs’ size to 40 nm AuNPs, proposing higher sensitivity from SeNPs compared to AuNPs due to larger particle size. We chose AuNPs of size 40 nm for comparison based on previous research that suggested that for AuNPs, although the detection signal increases with the particle size, the stability of the particle decreases with the size over 40 nm.^42^ Also, SeNPs maintain a bright color at larger sizes due to an intense peak in the light scattering component of their signal and therefore could fill a gap in the market that AuNPs could not unless modified.

## 2 Materials and Methods

### 2.1 Materials

A nitrocellulose (NC) membrane (UniSart, CN95) was purchased from Sartorius, Germany. An absorbent pad (A222) was purchased from Kenosha tapes, the Netherlands. A backing card of 60 mm width and 0.01 in. thickness was obtained from Lohmann, USA. The monoclonal mouse anti-IL-6 antibodies (L152 and L395) and recombinant human IL-6 antigen were purchased from Hytest Ltd., Finland. L152 was used as a capture antibody, and L395 was used as a detection antibody. Gold nanoparticles (AuNPs 40 nm, optical density 40) were purchased from Abcam, UK. Phosphate-buffered saline (PBS), Tris-buffered saline (TBS), casein, amicon filter units, hydroxylamine, and Tween-20 were purchased from Sigma-Aldrich, UK. Selenium dioxide (SeO_2_, 98%), sodium thiosulfate pentahydrate (NaTP, 99.5%), and sodium dodecyl sulfate (SDS, 98.5%) were all acquired from Sigma-Aldrich. Deionized water was produced at Glantreo Ltd.

### 2.2 Equipment

An Agilent 8453 UV–Vis spectrophotometer was employed to estimate the size of SeNPs both during their synthesis and post-centrifugation. A Beckman Coulter Avanti J-26 XPI centrifuge was used for centrifuging SeNP solutions. The average hydrodynamic diameter (*Z*-average) and polydispersity index (PDI) of selenium samples were measured by dynamic light scattering (DLS) using a Zetasizer (Nano ZS) from Malvern Instruments, a JEM-1400 Plus transmission electron microscope from JOEL was used for size characterization, a ZX1010 dispense platform from Biodot was used for antibody printing onto the NC membrane, and a Leelu reader (LUMOS-V3-03) from Lumos Diagnostics was used to analyze the test lines from the LFDs.

### 2.3 Selenium Nanoparticle (SeNP) Synthesis

SeNPs were synthesized using a multisolution chemical reduction method, in which SeO_2_ was reduced by NaTP in the presence of the particle stabilizer SDS. Initially, all glassware was washed with a mild detergent and rinsed with deionized water. Solution I was prepared by dissolving SDS and SeO_2_ in deionized water. Solution II was prepared by dissolving SDS and NaTP in deionized water. The concentration of SDS in solutions I and II was chosen to be well above its critical micelle concentration. The concentrations of SeO_2_ and NaTP in solutions I and II were varied such that the molar ratio of the selenium precursor to reducing agent was in the range of 0.51–0.69.

Both solutions I and II were placed into a temperature-controlled refrigeration unit held at 16 °C while being thoroughly mixed. Once both solutions were sufficiently mixed, solution II was promptly added to solution I to generate solution III. Solution III was left in the refrigeration unit while mixing, until the appearance of an orange/red color, which signified the formation of SeNPs.

Once the SeNPs were determined to be of the desired size, the SeNP solutions were washed by centrifugation. Briefly, the SeNP solutions were centrifuged until the appearance of a SeNP pellet at the base of the centrifuge tubes. The supernatant of the washed solution was discarded, and the SeNP pellet was resuspended using deionized water to a volume which corresponded to an optical density of 20 ± 1. The SeNP solutions were then stored at 16 °C.

### 2.4 Ultraviolet–Visible (UV–Vis) Spectrophotometry Characterization of SeNPs

The SeNP solutions were first diluted to optical density 1 using deionized water. Subsequently, 4 mL of aliquots was inserted into a polystyrene cuvette (path length = 1 cm) and analyzed over the wavelength range 190–1100 nm. The peak positions in the spectra of the SeNP solutions were converted to estimated sizes using an open-source computational model based on Mie extinction theory.^43^ All UV–Vis spectrophotometry measurements were performed in triplicate to ensure reproducibility.

### 2.5 Dynamic Light Scattering (DLS) Characterization of SeNPs

Measurements were carried out at 25 °C in triplicate, with each replicate consisting of 13 sub-measurements with 20 s acquisition time repeated 3 times (9 size values recorded and averaged per sample). The particles were illuminated with a helium–neon laser (633 nm), and the scattered light was collected at a back-scatter angle of 173°. Approximately 450 μL of the sample was inverted for 30 s, placed in a microcuvette (ZEN0040; Malvern Instruments), and equilibrated at 25 °C for 120 s prior to analysis. The refractive index and absorption coefficient were set at 1.590 and 0.010, respectively, for SeNPs. Results were reported from the intensity-based particle size distribution.

### 2.6 AuNP Conjugation

The IL-6 antibody (L395) was purified to remove amine molecules which can interfere with the conjugation process and resuspended in 10 mM PBS, pH 7.4. The AuNPs–IL-6 conjugate was prepared by incubating 20 μL of 1 mM EDC, 40 μL of 1 mM NHS, 10 μL of 150 mM MES buffer, and 20 μL of 0.1 mg/mL antibody to 50 μL of AuNPs, optical density 40 for 20 min. A hydroxylamine quencher (1 μL) was added, and the solution was incubated for a further 10 min. Then, 1 mL of TBS (containing 0.05% Tween) was added to the mixture and centrifuged at 3700 RCF for 10 min. The supernatant was removed, and the pellet was resuspended with 90 μL of 1× TBS (containing 0.5% casein and 0.05% Tween) to obtain an AuNPs–antibody conjugate of optical density 20.

### 2.7 SeNP Conjugation

Prior to SeNP conjugation, the optimal buffer pH was determined empirically since the isoelectric point differs between each antibody. An optimal pH of 7.4 was required for this conjugation. To conjugate the purified IL-6 detection antibody to SeNPs, 25 μL of 0.5 mg/mL antibody diluted in PBS was incubated with 250 μL of SeNPs, optical density 20 for 1 h. The mixture was then centrifuged for 1 h at 200 RCF, and the supernatant was removed. The pellet was resuspended with 250 μL of PBS (containing 0.5% casein + 0.1% Tween) to give a final SeNPs–antibody conjugate of optical density 20.

### 2.8 Fabrication of a Half Dipstick LFD

The capture antibody was diluted to 1 mg/mL in 1 mM phosphate buffer. The anti-IL-6 antibody (L152) was dispensed onto the NC membrane using a BioDot (ZX1010) dispensing platform at a flow rate of 1 μL/cm to obtain a test line width of 1 mm. Then, the NC membrane was dried in an oven at 37 °C for 1 h. The simplified half dipstick LFD was then assembled by placing the NC membrane on the backing card followed by the absorbent pad with a 5 mm overlap on the NC membrane. Finally, strips were cut 5 mm wide before use.

### 2.9 Transmission Electron Microscopy Characterization

A JOEL JEM-1400 Plus transmission electron microscope was used at 120 kV acceleration voltage.

## 3 Results and Discussion

**Figure 1:**
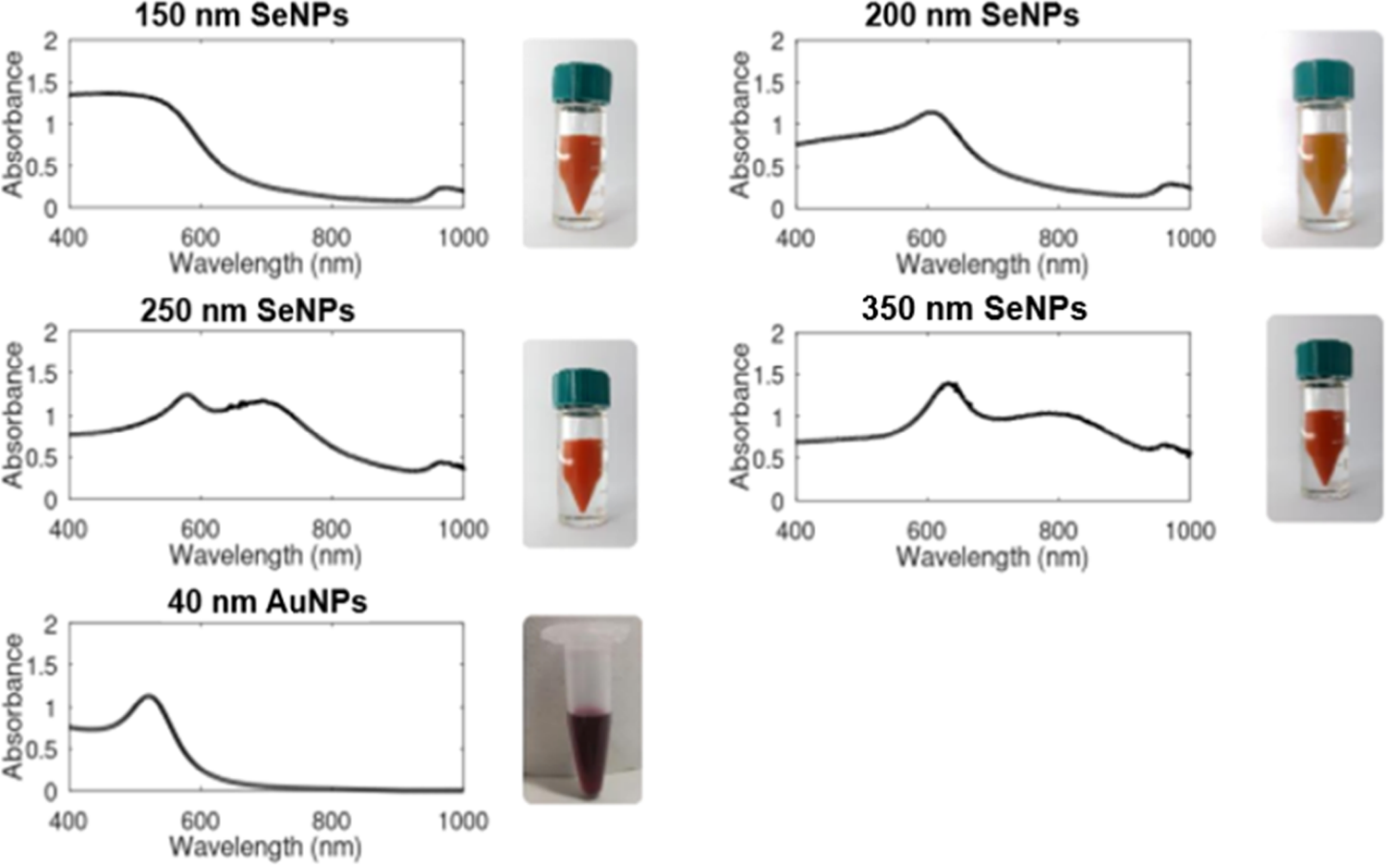
(Above: top to bottom) UV–Vis spectra of 150, 200, 250, and 310 nm SeNP solutions and 40 nm AuNPs. All UV–Vis spectra were recorded at an optical density of 1 using deionized water, with air as the blank. Photographs of colloid solutions at OD 20 ± 1 are shown to the right of each spectrum. SeNP photograph courtesy of M.A.C. and AuNP photograph courtesy of Z.B. Copyright 2022.

Figure 1 shows images of 150, 200, 250, and 310 nm sized SeNPs that were produced using a multisolution chemical reduction method along with their ultraviolet–visible (UV–Vis) spectrophotometry spectra. As the SeNPs increase in size, the solution color darkens and shifts to red as previously reported by Lin et al.^24^ and Gangadoo et al.^25^

As the size of SeNPs increases, the UV–Vis spectrum shows that an additional peak appears and shifts the existing spectral peak to longer wavelengths.^44,45^ This is most clearly observed between the 200 and 250 nm sized SeNP solutions where the emergence of a second peak at 580 nm corresponds with the shift of the first spectral peak from wavelength 606 to 696 nm, aligning with the experimental observations of Lin et al.^24^ and the computational predictions of Dauchot et al.^45^

**Figure 2:**
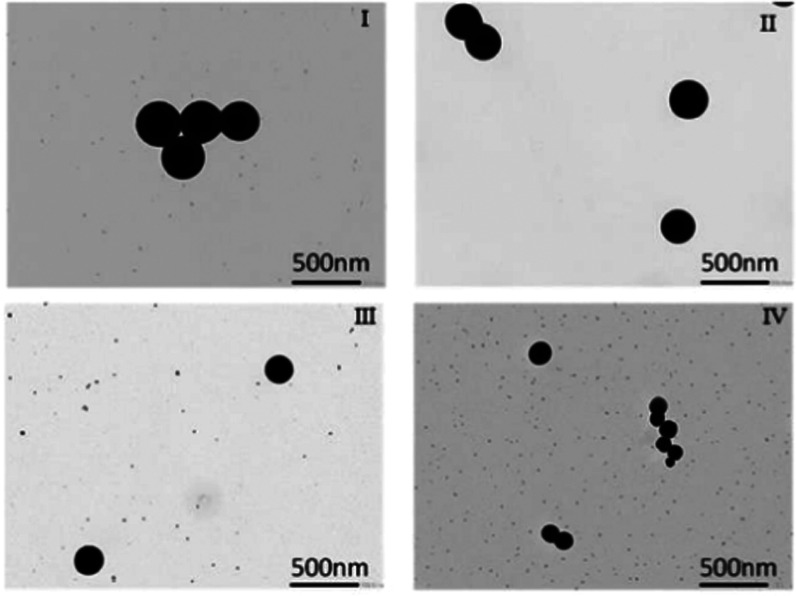
TEM images of SeNP labels employed in the detection of IL-6. Images (I–IV) correspond to 310, 250, 200, and 150 nm respectively. Images were recorded at ×20 000 magnification.

From Table 1, the presented Mie sizes were calculated using Mie extinction theory.^45−47^ The DLS sizes are the averages from nine respective measurements, while the recorded PDIs are the maximum values from the said nine measurements. SeNPs were also characterized through dynamic light scattering (DLS) and transmission electron microscopy to directly examine their morphology. TEM images of SeNPs are presented in Figure 2 I–IV. DLS size estimates for the four SeNP labels considered here are presented in Table 1, with size distribution plots presented in Figures S1–S4. All employed SeNPs are spherical as seen in the TEM images, as well as highly monodisperse with maximum polydispersity index (PDI) values below 0.1 (see Table 1).^48^ Characteristics are a result of the employed chemical reduction synthesis method, which is known for producing size-controlled monodisperse SeNPs.^24,25^ Note that while the recorded DLS size estimates presented here are larger than the listed sizes which were determined using Mie theory,^45−47^ this can be accounted for by considering that DLS measures the hydrated size of NPs,^48^ resulting in a slight overestimation compared to the predicted Mie SeNP sizes outlined here.

**Table 1:** Predicted Mie Size, DLS Average Size, and Maximum Polydispersity Index (PDI) Values for the 150, 200, 250, and 310 nm SeNP Labels

Mie size (nm)	DLS average size (nm)	maximum PDI
310	323	0.07
250	266	0.05
200	249	0.08
150	163	0.09

**Figure 3:**
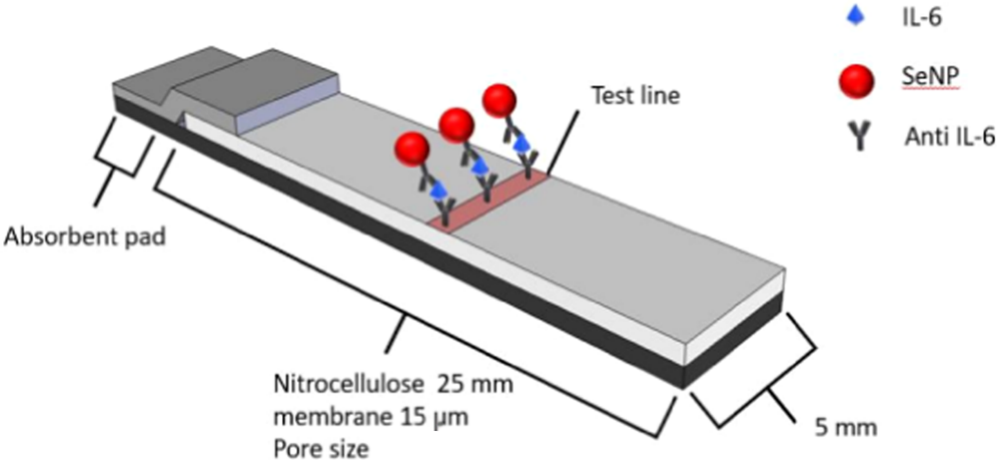
Schematic illustration of the SeNP-based half dipstick LFDs employed in the detection of IL-6. Note the accumulation of SeNPs at the test area upon conjugation with the analyte IL-6 and anti-IL-6 antibodies, resulting in the formation of a colored line.

Once characterized and conjugated, all NPs were employed in half dipstick LFDs for the detection of the sepsis biomarker IL-6 (Figure 3). Briefly, the conjugate pad was coated with an anti-IL-6 antibody functionalized with either SeNPs or AuNPs, while the test line was coated with an unfunctionalized anti-IL-6 antibody. Upon the introduction of a sample containing IL-6, the SeNP- or AuNP-functionalized anti-IL-6 antibody bound to the IL-6 analyte. Upon traversing the membrane region of the LFD, the IL-6/anti-IL-6/SeNPs or IL-6/anti-IL-6/AuNPs conjugate was captured at the test area creating a test line, the color and intensity of which are dependent on the size and type of NPs employed and proportional to the quantity of NPs captured at the test line as well as the concentration of IL-6 in the sample.

**Figure 4:**
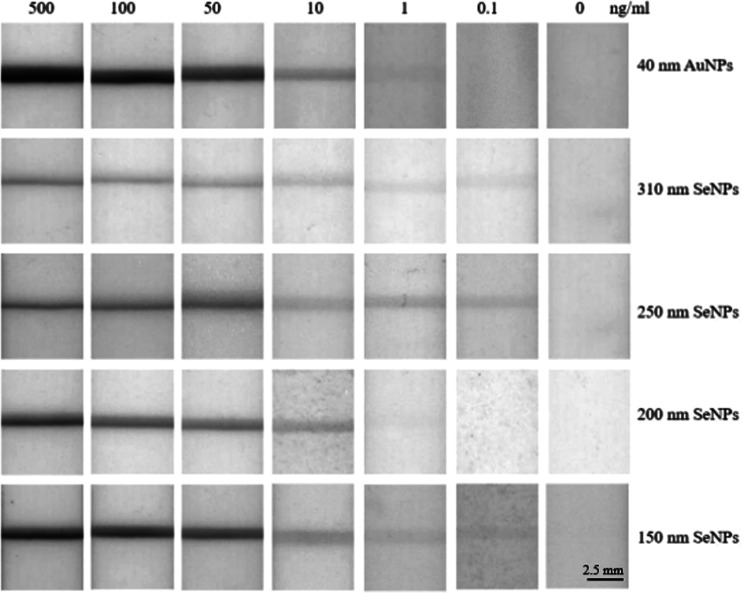
LFDs investigating the sensitivity of 40 nm AuNPs and 150–310 nm SeNPs toward IL-6 in the concentration range 0–500 ng/mL. The intensity of the resulting colored test lines was quantified through ImageJ analysis software. Each LFD measurement was performed in duplicate to ensure reproducibility.

**Figure 5:**
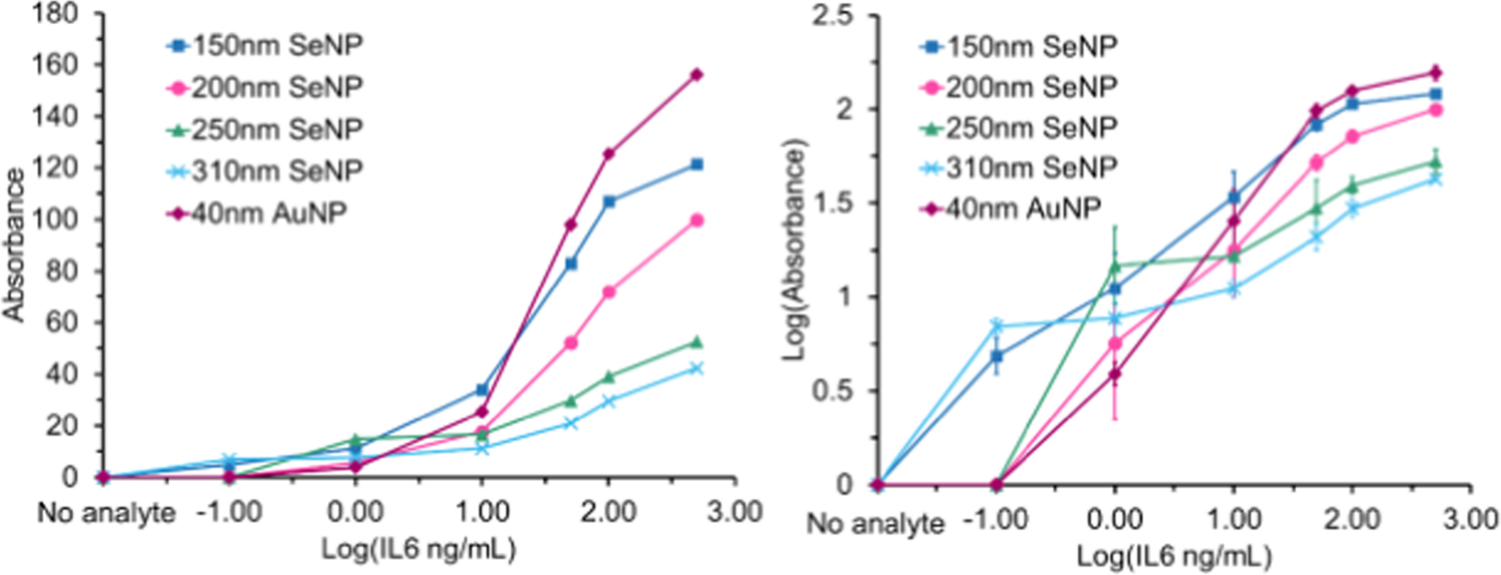
Left: Test line absorbance vs log 10 IL-6 concentration plot for each SeNP- and AuNP-based LFD. Right: Log 10 test line absorbance vs log 10 IL-6 concentration plot for each SeNP- and AuNP-based LFD. The intensity of the test line is measured by grayscale, while the concentration of IL-6 is presented in ng/mL as above. For numerical values used in the construction of the above plot, see Table S1.

Upon constructing LFDs based on SeNPs and AuNPs, a series of IL-6-spiked buffer solutions with concentrations between 0.1 and 500 ng/mL were utilized to evaluate the sensitivity of the LFDs as a function of NP size (Figure 4). For direct images of the employed LFDs, see Figure S5. Based on visual inspection, the 40 nm AuNPs followed by the 150 nm SeNPs produced the greatest intensity test lines between 10 and 500 ng/mL IL-6, while the 310 nm SeNPs followed by 150 nm SeNPs provided the greatest intensity test lines at 0.1 ng/mL IL-6. To quantify the intensity of the test lines produced by each sized SeNPs and AuNPs at the IL-6 concentrations considered here, ImageJ analysis software was employed to construct a plot of test line absorbance vs IL-6 concentration, as shown in Figure 5.

Consistent with the visual inspection observations discussed above, the largest- and smallest-sized SeNPs provide the greatest levels of sensitivity at low concentrations of IL-6 (0–1 ng/mL). The 310 and 150 nm sized SeNPs both report an LOD of 0.1 ng/mL, compared to the 200 nm, 250 nm SeNPs, and 40 nm AuNPs which record an LOD of 1 ng/mL (see Figure S6 for an explicit plot of the LOD for each label). At moderate to high concentrations of IL-6 (1–500 ng/mL), the smaller 40 nm AuNPs and 150 nm SeNPs produce a more intense band, with both recording intensity values 3 times that of the 310 nm SeNPs. As such, the larger-sized SeNPs record a low detection limit at the cost of signal intensity at high concentrations of IL-6. Conversely, 200 and 250 nm SeNPs, as well as 40 nm AuNPs, provide poorer detection limits, with the latter producing more intense test lines at high concentrations of IL-6.

The reduced sensitivity with increasing IL-6 concentration for the larger-sized SeNPs can be explained by considering both the prozone effect and inherent steric hindrance effects. While larger NPs have a larger surface area and more binding sites per particle, they have a lower surface area-to-volume ratio than their smaller counterparts. Therefore, there are fewer antibody binding sites on larger NPs than on the equivalent volume of smaller NPs. At low concentrations of IL-6, 310 nm SeNPs performed better as they are physically bigger and fewer binding events are required to produce a visible band. However, as the analyte concentration increases, fewer larger SeNPs can bind at the test line due to steric hindrance preventing close packing. At very high concentrations of IL-6, the larger SeNPs become saturated with IL-6 due to fewer supporting antibodies and excess IL-6 binds to antibodies at the test line. This prevents larger SeNPs from forming sandwich complexes and decreases the accumulation of NP–analyte conjugates causing test line intensity to decrease.^40^ Additionally, larger-sized SeNPs may struggle to traverse the membrane section of the LFD if the pores are too small to accommodate the large NP–analyte conjugates, again resulting in the poor accumulation of large SeNPs at the test area.^49^

The higher LOD reported for the 200 and 250 nm SeNPs relative to their 150 and 310 nm counterparts is believed to be due to a suboptimal combination of optical intensity and steric hindrance effects. While the 150 nm SeNPs lack the intense optical profile of their larger counterparts, they are relatively unaffected by steric hindrance effects while having a larger number of available binding sites per sample volume, thus allowing the production of an intense band at both low and high IL-6 concentrations. Conversely, the 310 nm SeNPs suffer from steric hindrance effects, but due to their darker optical profiles, they record a lower LOD. However, it is believed that the 200 and 250 nm SeNPs suffer from both relatively weak optical profiles and steric hindrance effects, resulting in their high LOD and low band intensities at elevated IL-6 concentrations.

The 150 nm SeNPs combined the sensitivity of the large 310 nm SeNPs with a low LOD of 0.1 ng/mL IL-6 and the 40 nm AuNPs’ visual intensity of test lines into one product. Since the early detection of IL-6 is of greater significance than its detection over a wide concentration range, i.e., in the diagnosis of neonatal sepsis, the 150 nm SeNPs offer a more desirable detection profile than the 40 nm AuNPs tested here.

It should also be noted that none of the NPs tested here displayed nonspecific binding, thus highlighting the excellent specificity of all of the NPs tested and the robustness of the employed methods for both conjugating each respective NP and constructing the LFDs.^50,51^

To obtain even lower LOD than those reported here, even larger SeNPs should be investigated to determine if their darker optical profiles overcome their associated steric hindrance limitations. However, the upper size limit of the SeNPs will be restricted by the pore size of the membrane in the LFD as previously mentioned. Additionally, by increasing the size of the employed NPs, the risk of nonspecific binding events taking place increases.^38,52^ Thus, increasing the size of the SeNPs would require further method development and optimization of LFD parameters.

## 4 Conclusions

In conclusion, SeNPs of sizes 150, 200, 250, and 310 nm were synthesized through a multisolution chemical reduction method and characterized through UV–Vis spectrophotometry, DLS, and TEM. All SeNPs were demonstrated to be monodisperse and spherical in morphology.

The SeNPs were utilized in LFDs along with 40 nm AuNPs for the detection of the biomarker IL-6. The 310 and 150 nm sized SeNPs recorded the lowest LOD of 0.1 ng/mL, while the 40 nm AuNPs displayed the highest visual intensity at elevated IL-6 concentrations. The drop-off in sensitivity with increasing IL-6 concentration for larger SeNPs is hypothesized to be due to both the prozone effect and steric hindrance limitations, emphasizing the importance of investigating which sized label is the most effective for a specific LFD application.^39,51^

The 150 nm sized SeNPs are proposed as the best all-around label for the detection of IL-6, offering the lowest LOD of 0.1 ng/mL and comparable visual detection to 40 nm AuNPs at high analyte concentrations. The lower LOD offered by 150 nm SeNPs over 40 nm AuNPs is especially desirable in applications where the early detection of an illness is critical to the speed with which targeted treatment can be provided, e.g., the early detection and treatment of sepsis in primary care settings.

SeNPs have been demonstrated as a viable alternative to AuNPs for use in future commercial POC diagnostic devices for the detection of sepsis. Future work would involve further optimization of large SeNPs to improve sensitivity and investigate the cross-reactivity of IL-6 for assay specificity. Once the assay is optimized, trials with patient blood samples can be carried out to compare performance to other IL-6 LFDs in the detection of sepsis. For future research, we aim to test AuNPs against SeNPs of identical size for a direct comparison of selenium and gold.

## Supporting Information Available

Materials, experimental methods, photographs of SeNP and AuNP LFDs, DLS results for synthesized SeNPs, and numerical absorbance values for each LFD test line (PDF)

## Notes

J.P.H., M.A.C., J.M., and J.H. are all employees of Glantreo Ltd. which manufactures SeNPs for commercial use. Z.B., S.F., J.A.M., and N.B. work at Ulster University. Z.B. works in conjunction with Biopanda Reagents Ltd. which manufactures lateral flow kits for commercial use. T.U. works at University College Cork and P.A.C. works in conjugation with Glantreo Ltd. and University College Cork. K-T.Y. and P.M. work at Biopanda Reagents Ltd.

## Abbreviations

LFDs: lateral flow devices
POC: point-of-care
IL-6: interleukin-6
LXNPs: latex nanoparticles
AuNPs: gold nanoparticles
SeNPs: selenium nanoparticles
LOD: limit of detection
UV–vis: ultraviolet–visible
DLS: dynamic light scattering
