# Supplementary material for: Effect of Selenium Nanoparticle Size on IL-6 Detection Sensitivity in a Lateral Flow Device
Source: ACS Omega. 2023 Feb 23;8(9):8407–14. doi: 10.1021/acsomega.2c07297 (PMC9996617; doi:10.1021/acsomega.2c07297)
Supplement: Supplementary file 1 — ao2c07297_si_001.pdf [file ao2c07297_si_001.pdf]

Effect of Selenium Nanoparticle Size on IL-6 Detection Sensitivity in a Lateral Flow Device

[3,4]Zoe Bradley, [1,2]Patrick A. Coleman, [1]Melissa A. Courtney, [,4]Sam Fishlock, [1]Joseph McGrath, [6]Therese Uniacke-Lowe, [4,5]Nikhil Bhalla, [4]James A. McLaughlin, [1]John Hogan, [1]John P. Hanrahan, [3]Ke-Ting Yan and [3]Philip McKee.*

[1] Glantreo Ltd., Environmental Research Institute, Cork, T23 XE10, Ireland.

[2] Department of Chemistry, College of SEFS, University College Cork, Kane Building, Cork T12 YN60, Ireland.

[3] Biopanda Reagents Ltd., Unit 14 Carrowreagh Business Park, Carrowreagh Road, Belfast, BT16 1QQ, United Kingdom.

[4] Nanotechnology and Integrated Bioengineering Centre, School of Engineering, University of Ulster, Belfast, BT15 1ED, United Kingdom.

[5] Healthcare Technology Hub, School of Engineering, University of Ulster, Belfast, BT15 1ED, United Kingdom.

[6] Department of Chemistry, School of Food and Nutritional Sciences, University College Cork Leve 2 Food Science Building, Cork T12 TP07, Ireland.

[*] Corresponding Author: z.woods@ulster.ac.uk

Presented within this supporting information document are the following sections:

[1] Supplementary Figures S1 – S6

[2] Supplementary Table S1

1. Supplementary Figures

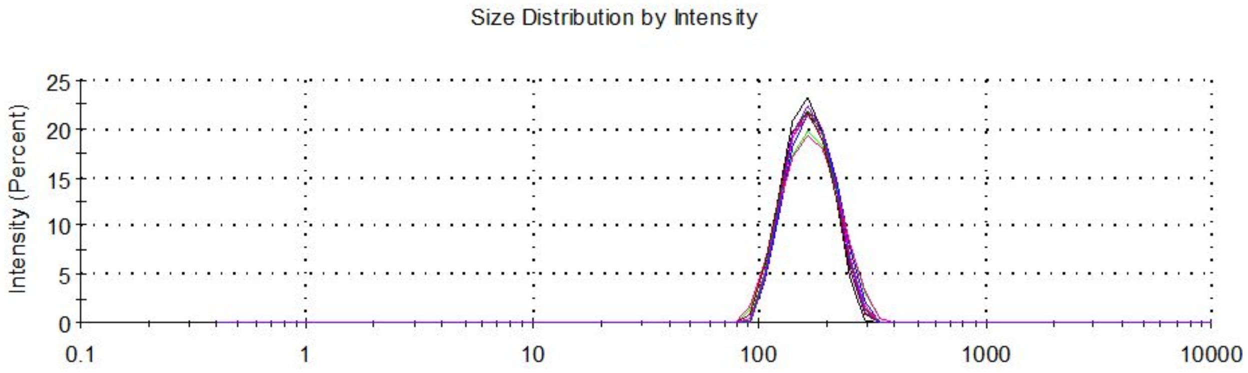

Figure S1: Size distribution plot of the nine recorded DLS measurements performed on the 150 nm SeNPs. The average diameter from the above size distribution plot is presented in Table 1 in the main document.

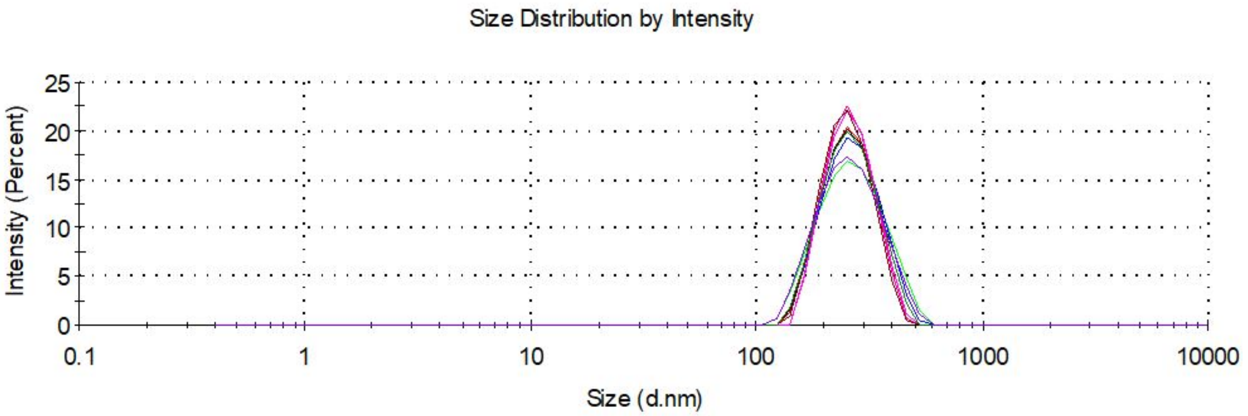

Figure S2: Size distribution plot of the nine recorded DLS measurements performed on the 200 nm SeNPs. The average diameter from the above size distribution plot is presented in Table 1 in the main document.

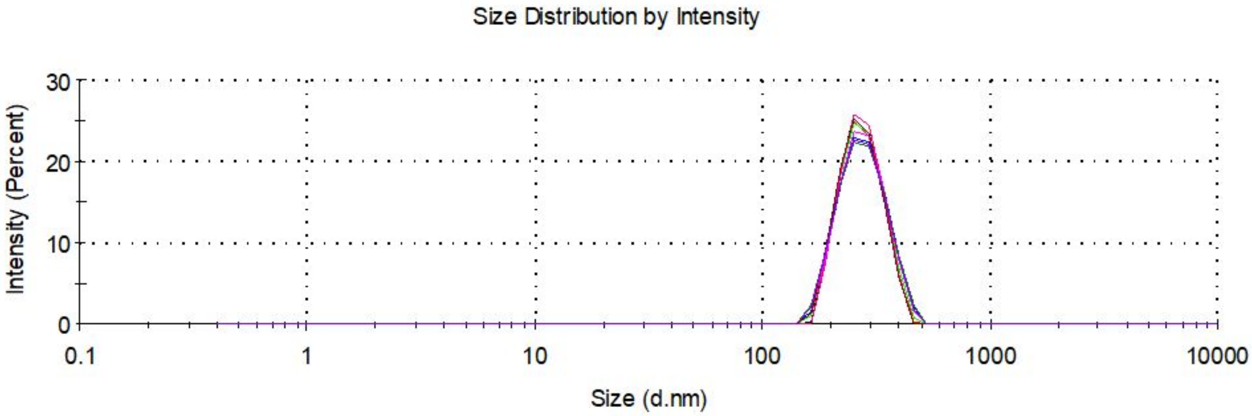

Figure S3: Size distribution plot of the nine recorded DLS measurements performed on the 250 nm SeNPs. The average diameter from the above size distribution plot is presented in Table 1 in the main document.

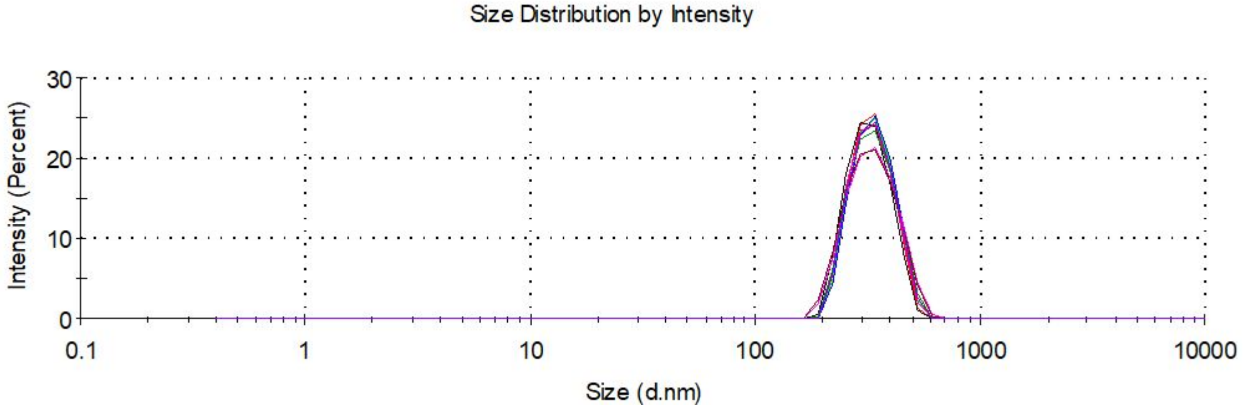

Figure S4: Size distribution plot of the nine recorded DLS measurements performed on the 310 nm SeNPs. The average diameter from the above size distribution plot is presented in Table 1 in the main document.

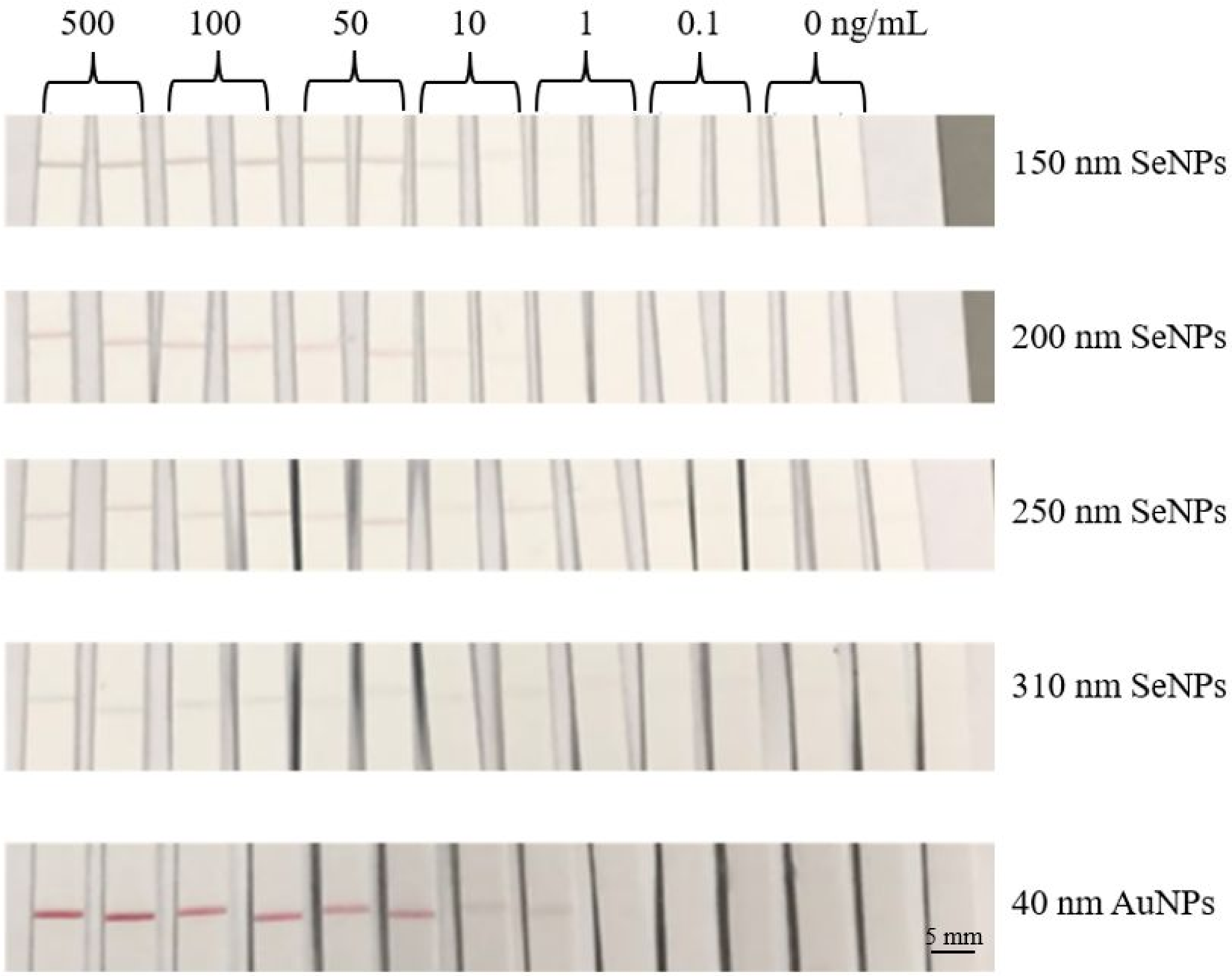

Figure S5: Direct images of the SeNPs and AuNPs-based LFDs used in the investigation of IL-6 sensitivity. **(Left to right)** LFDs were subjected to successively lower concentrations of IL-6. **(Top to bottom)** LFDs which employed successively larger SeNPs, and finally AuNPs. All LFDs were analyzed in duplicate to ensure reproducibility.

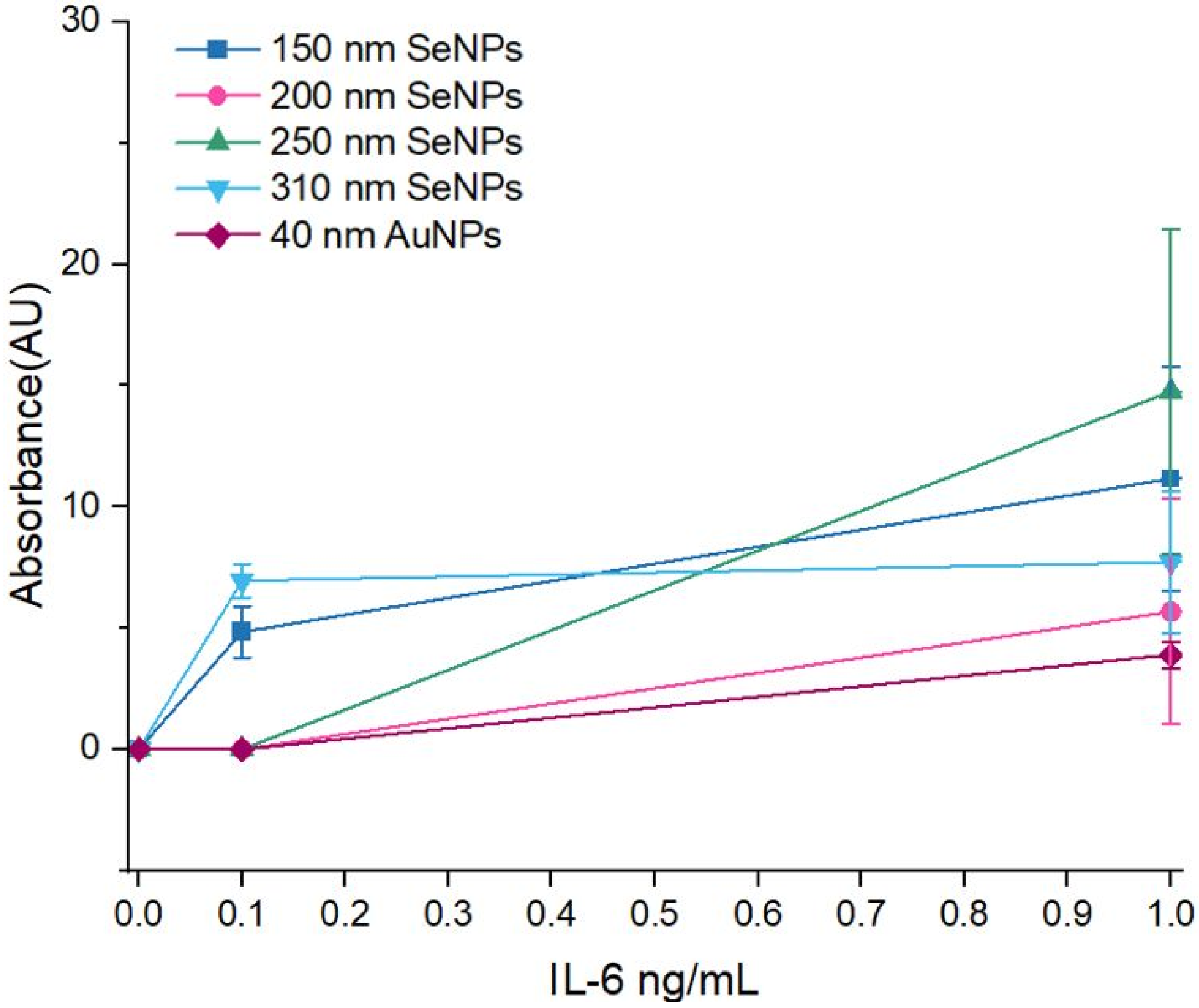

Figure S6: Absorbance vs IL-6 concentration plot for each SeNPs and AuNPs-based LFD. The above plot illustrates the LOD of each label. Specifically, 0.1 ng/ml for the 150 nm and 310 nm sized SeNPs labels, and 1 ng/ml for the 200 nm and 250 nm sized SeNPs labels as well as the 40 nm AuNPs label.

2. Supplementary Table

Table S1: Sensitivity values run in duplicate for the 150 nm, 200 nm, 250 nm, 310 nm SeNPs and 40 nm AuNPs-based LFDs in the detection of IL-6. Values were recorded using Image J analysis software.

IL-6 (ng/mL)	150 nm SeNPs		200 nm SeNPs		250 nm SeNPs		310 nm SeNPs		40 nm AuNPs	
**500**	122	121	102	98	47	58	43	42	166	146
**100**	110	104	68	76	36	42	27	32	128	123
**50**	88	79	48	56	23	37	19	23	104	93
**10**	41	27	25	11	16	17	12	10	32	19
**1**	14	8	9	2	10	20	10	6	4	4
**0.1**	6	4	0	0	0	0	7	6	0	0
**0**	0	0	0	0	0	0	0	0	0	0
